# Transmembrane domain is crucial to the subcellular localization and function of Myc target 1

**DOI:** 10.1111/jcmm.12747

**Published:** 2015-12-29

**Authors:** Shuai Wu, Jinghua Gui, Xiaofei Yin, Qiang Pan, Xinyuan Liu, Liang Chu

**Affiliations:** ^1^ State Key Laboratory of Cell BiologyInstitute of Biochemistry and Cell BiologyShanghai Institutes for Biological SciencesChinese Academy of SciencesShanghaiChina; ^2^ Dr. Gui, Institute of BiotechnologyUniversity of HelsinkiHelsinkiFinland

**Keywords:** MYCT1, transmembrane domain, subcellular localization, cell migration

## Abstract

Deregulation of c‐MYC occurs in a variety of human cancers. Overexpression of c‐MYC promotes cell growth, proliferation, apoptosis, transformation and genomic instability. MYC target 1 (MYCT1) is a direct target gene of c‐MYC, and its murine homologue MT‐MC1 recapitulated multiple c‐Myc‐related phenotypes. However, the molecular mechanism of MYCT1 remains unclear. Here, we identified the transmembrane (TM) domain of MYCT1, not the nuclear localization sequence, is indispensable to the vesicle‐associated localization of MYCT1 protein in the cytoplasmic membrane vesicle. Overexpression of MYCT1, not MYCT1 (ΔTM), decreased cell viability under serum deprivation and increased tumour cell migration ability. We further identified CKAP4 interacted with MYCT1 and contributed to the function of MYCT1. In addition, we found that a mutation, A88D, which is observed in patient sample, changed the localization, and abolished the effect on cell viability and cell migration, suggesting that the TM domain is critical to MYCT1.

## Introduction

C‐myc is an oncogene, and is frequently activated in a broad range of human cancers. Deregulation of c‐MYC often associates with aggressive and poorly differentiated tumours. C‐MYC protein mainly functions as a transcription factor and regulates a variety of cellular processes including cell growth, proliferation, apoptosis, cell differentiation, transformation and genomic instability 1, 2, 3, 4. It is reported that c‐MYC regulates over 15% of genomic genes through binding to the consensus sequence termed E‐boxes 5, 6, 7. The target genes are involved in many intracellular signalling pathways. Therefore, it is difficult to explore which gene contributes to specific c‐MYC‐associated phenotype, and only a small number of genes were distinguished. For example cyclin B1 induces tetraploidy 8. Ornithine decarboxylase mediates c‐MYC‐induced apoptosis 9. CDK4 controls cell cycle progression 10. However, these genes can only recapitulate limited phenotypes of c‐MYC.

MYCT1, a direct target gene of c‐MYC, was first cloned from laryngeal squamous cell carcinoma (LSCC) cells. Overexpression of murine MYCT1 (MT‐MC1) rescues multiple phenotypes of c‐Myc knockout mouse cell lines 11, 12. MT‐MC1 affects several cellular processes including cell cycle progression and apoptosis, cell transformation, cell differentiation and genomic instability 13. Yin *et al*. analysed the sequence of MT‐MC1 protein and found a low conserved domain of a DNA helicase of virus and a putative nuclear localization sequence (NLS) 13. Rogulski *et al*. identified 47 genes deregulated by MT‐MC1 using transcriptional profiling 11. However, they did not find DNA binding domain in MT‐MC1 protein or consensus DNA binding sequences. How MT‐MC1 regulates its target genes and mimics most c‐MYC phenotypes remains unclear.

The expression of MYCT1 is detectable in various tissues. Certain types of tumour tissues, such as gastric carcinoma and LSCC tissue, have lower MYCT1 expression compared to normal tissues 14, 15. Recently, it was reported that down‐regulation of MYCT1 may be involved in LSCC development and invasion, and overexpression of MYCT1 significantly decreases cell viability and the invasive ability of the LSCC cell line Hep2 cells. However, MYCT1 overexpression did not trigger programmed cell death in HEK293 cells 15.

In this study, we analysed the protein sequence of MYCT1 and proved that the second transmembrane domain (TM) determined its granularly cytoplasmic localization and function. We identified that CKAP4 was a MYCT1 interacting protein, and contributed to MYCT1‐mediated cell migration. In addition, we demonstrated that the point mutation (A88D), which exists in a clinical cancer sample, changed the localization and function of MYCT1.

## Materials and methods

### Cell culture and reagents

HeLa cell and HEK293T cell were cultured with DMEM (Invitrogen, Carlsbad, CA, USA) supplemented with 10% foetal bovine serum (FBS; Biochrom, Berlin, Germany). SW480 cell was cultured with Leibovitz's L‐15 (Invitrogen) supplemented with 10% FBS. A549 cell was cultured with F12K (Invitrogen) supplemented with 10% FBS. HT‐29 cell was cultured with McCoy's 5A (Invitrogen) supplemented with 10% FBS. All the cells were maintained at 37°C in an atmosphere of 5% CO_2_, 95% air. C‐MYC and MYCT1 cDNAs were purchased from Proteintech Group (Wu Han, China). STX6 cDNA was donated by Professor Jiahuai Han. Etoposide was purchased from Sigma‐Aldrich (St. Louis, MO, USA). Human recombinant epidermal growth factor (EGF) was purchased from BD Biosciences (Franklin Lakes, NJ, USA). Falcon^™^ Cell Culture Inserts were purchased from BD Biosciences. Anti‐Flag (M20008), His (M20001) and Actin (M20009) antibodies were purchased from Abmart (Shanghai, China). Anti‐β‐catenin (E‐5) antibody was purchased from Santa Cruz Biotechnology (Santa Cruz, TX, USA). Anti‐p‐AKT (Ser473), AKT antibody, anti‐p‐GSK (Ser9) antibody and Vesicle Trafficking Antibody Sampler Kit were purchased from Cell Signaling Technology (Danvers, MA, USA). Anti‐TCF‐4 (EP2033Y) antibody was purchased from Millipore (Billerica, MA, USA). DAPI was purchased from Beyotime (Jiangsu, China). Alex Fluor 488 and 555 conjugated secondary antibodies were purchased from Life Technologies (Carlsbad, CA, USA).

### Plasmids construction

MYCT1 promoter (−925 bp/−145 bp) was cloned from HeLa genome by KOD DNA polymerase (TOYOBO, Osaka, Japan) and cloned into pGL3 vector (Clontech, Mountain View, CA, USA). Two primers contain KpnI and BglII restriction enzyme sites, respectively, and their sequences are as follows: primer −925: 5′‐TAGGTACCTATTTATGAAATGAATTAAATAACTTTC‐3′; and primer −145: 5′‐GCAGATCTAAAAGGAAATAAGTGTATCATGTTTCCT‐3′. For cloning of GFP‐fused truncation mutants, GFP sequence was cloned at the end of the truncation mutants by overlap PCR. Deletion mutations or point mutations were also constructed by overlap PCR. These sequences were all inserted into the BamHI/EcoRI site of pLVX‐Puro or pLVX‐flag‐Puro lentivirus plasmid vector (Clontech).

### Lentiviral package and infection

For lentivirus package, HEK293T cell was transfected by Effectene (Qiagen Hilden, Germany) according to the manufacturer's protocol. For stable transfection, cells were infected with lentiviruses and were selected by incubation with 1–2 μg/ml puromycin beginning at 48 hrs post‐infection.

### Luciferase reporter assay

Cells in 48‐well plates were transfected in quintuple using Effectene. For MYCT1 promoter assay, the pGL3‐MYCT1 promoter, pcDNA3‐c‐MYC and the Renilla luciferase plasmid (Promega, Madison, WI, USA) were cotransfected into HEK293T cells for 24 hrs with 50 ng, 50 ng and 2 ng respectively. For Wnt signalling pathway reporter TOPflash assay, SW480 cells, SW620 cells and HCT116 cells were transfected using Lipofectamine 2000 (Invitrogen) with 100 ng TOPflash and 2 ng Renilla luciferase plasmid respectively. HEK293T cells were transfected with 50 ng of TOPflash and 2 ng of Renilla luciferase plasmid together with 100 ng of β‐catenin and 100 ng of MYCT1 expression plasmids for 24 hrs. Luciferase assay was performed with the Dual Luciferase Assay System (Promega). Relative luciferase activity was calculated as the ratio of sample luciferase activity to Renilla luciferase activity.

### Western blot and immunoprecipitation

Whole cell lysates were prepared using radioimmune precipitation assay buffer or immunoprecipitation (IP) lysis buffer (Beyotime) and centrifuged at 13,500 × *g* for 10 min. at 4°C. For IP, the supernatant was incubated with a specific primary antibody overnight at 4°C in addition to A/G agarose (Roche, Basel, Switzerland). The beads were washed five times and resuspended in 60 μl SDS loading buffer. The samples were size‐fractionated by 10% SDS‐PAGE. The blots were incubated with primary antibodies. After incubation with secondary antibodies, the immunocomplexes were developed using chemiluminescence. For the IP assay, target protein was immunoprecipitated and washed six times with ice‐cold PBS before boiling in SDS loading buffer.

### RT‐PCR and Quantitative PCR

For RT‐PCR, total RNA was isolated using the TRIzol reagent (Invitrogen) and was used for RT‐PCR with the ReverTra Ace qPCR RT kit (TOYOBO). Quantitative PCR analysis was performed with the Bio‐Rad CFX96 Real‐Time PCR Systems. The following primers were used: AXIN2‐F (5′‐ATGCGTGGATACCTTAGACTTC‐3′) and AXIN2‐R (5′‐TCTGCTGCTTCTTGATGCC‐3′; c‐MYC‐F (5′‐CCTGGTGCTCCATGAGGAGAC‐3′) and c‐MYC‐R (5′‐CAGACTCTGACCTTTTGCCAGG‐3′); CCND1‐F (5′‐TCTACACCGACAACTCCATCCG‐3′) and CCND1‐R (5′‐TCTGGCATTTTGGAGAGGAAGTG‐3′); DKK1‐F (5′‐TCCCCT‐GTGATTGCAGTAAA‐3′) and DKK1‐R (5′‐TCCAAGA‐GATCCTTGCGTTC‐3′); SFRP1‐F (5′‐TCAGATTTCAACTCGTTGTCACAG‐3′) and SFRP1‐R (5′‐AGATGCTTAAGTGTGACAAGTTCC‐3′); MYCT1‐F (5′‐CACAACAAGTTTAGGGAGTCCATG‐3′) and MYCT1‐R (5′‐GCTGGAAGGTGAGACTGG‐3′); GAPDH‐F (5′‐GTCTCCTCTGACTTCAACAGCG‐3′) and GAPDH‐R (5′‐ACCACCCTGTTGCTGTAGCCAA‐3′).

### Immunofluorescence

Immunofluorescence was performed as described previously 16. Briefly, the cells were fixed with 4% polyformaldehyde, permeabilized with 0.5% Triton X‐100 and incubated in 5% BSA for 1 hr. The samples were incubated with the primary antibody overnight at 4°C and subsequently incubated with a secondary antibody conjugated to Alexa Fluor 595 or Alexa Fluor 488 (Life Technologies) for 1 hr at room temperature. Images were photographed and analysed using a Leica SP8 microscope (Wetzlar, Germany) equipped with a 63 × objective.

### GST pull‐down and mass spectrometry

GST‐MYCT1 protein with His‐tag was expressed by pGEX‐4T‐1 vector in BL21 bacteria. Cells were lysed in the appropriate volume of lysis buffer (50 mM Tris‐HCl pH 7.5, 150 mM NaCl, 10% glycerol, 1 mM DTT, 0.5% TritonX‐100, 2 mM MgCl_2_, 100 μg/ml with protein inhibitor cocktail). The lysis was pre‐purified with Ni‐NTA beads and eluted by 200 mM imidazole and then was re‐purified with GST beads. GST beads containing purified GST‐tagged protein were incubated with HeLa cell lysate (fifteen 10 cm‐plates) at 4°C overnight. Beads were washed five times with lysis buffer. The complex was subjected to SDS‐PAGE and coomassie staining. Specific bands were analysed by mass spectrometry (MS). LTQ‐VELS MS was performed by Shanghai Applied Protein Technology Company (Shanghai, China). Database searching was performed with a UniProt database selected for *Homo sapiens*.

### Cell viability and migration assay

Cell viability was assessed by the MTT colorimetric assay. For normal medium, HeLa stable cells were seeded 2000 per well in 96‐well plates. For serum deprivation medium or chemotherapy drug containing medium (etoposide, 5 μg/ml), about 8000 per well cells were seeded in 96‐well plate. At different time‐points (0, 1, 2, 3, 4 and 5 days), 20 μl of MTT (Sigma‐Aldrich) solution (5 mg/ml) was added into each well. Cells were incubated at 37°C for 4 hrs. The medium was replaced with 150 μl of DMSO and the plate was allowed to shake on a plate shaker for 15 min. Absorbance at 595 and 650 nm were measured using a microplate reader (Thermo Scientific, Waltham, MA, USA). Each group was repeated for 6 times. Values are expressed as fold changes *versus* empty vector (EV) group.

For cell migration assay, 5 × 10^4^ cells were resuspended in 500 μl serum‐free medium containing 100 ng/ml EGF and seeded to the 24‐well Falcon^™^ Cell Culture Inserts. 700 μl medium containing 10% FBS was added to the lower chambers. After incubation at 37°C for 24 hrs, cells were fixed with methanol containing 2% crystal violet. Cells that migrate to the underside were counted.

### Statistical analysis

All the data were shown as mean ± S.D. Comparison between two groups were performed by Student's *t*‐test using R software.

## Results

### Characterization of MYCT1 protein

**Figure 1:**
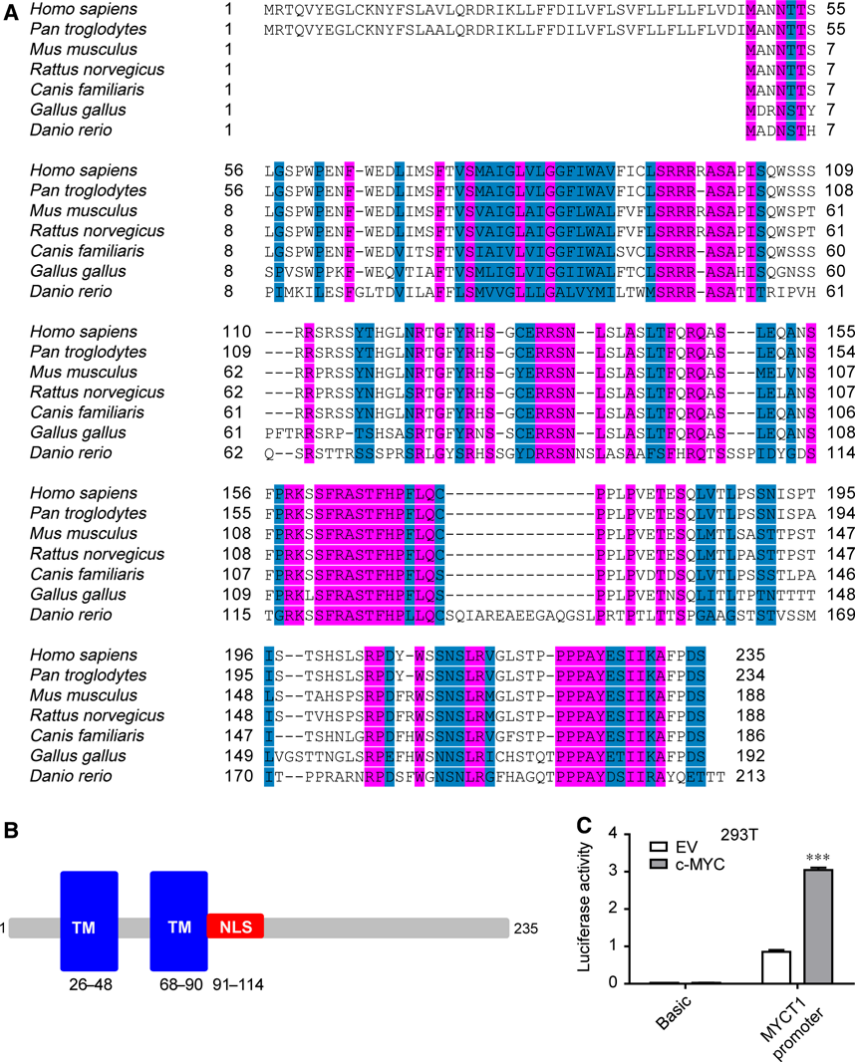
Sequence analysis of MYCT1 protein. (**A**) Cross‐species comparison of MYCT1 protein. All sequences were downloaded from NCBI and aligned by DNAssist 2.2 software. (**B**) Protein motif analysis of MYCT1 predicted by SMART, Pfam and CBS Prediction Servers. (**C**) MYCT1 promoter was regulated by c‐MYC. HEK293T cells were cotransfected with MYCT1 promoter reporter plasmid and pcDNA3‐c‐MYC. Luciferase activity was measured 24 hrs post‐transfection. EV: empty vector. Basic indicates pGL3Basic. ***P < 0.001, *versus* group of MYCT1 promoter with EV.

MYCT1 is evolutionarily conserved from zebrafish to human except fruitfly, which has no MYCT1 homologues. Only human and chimpanzee MYCT1 have additional 48 amino acids in the N‐terminus (Fig. 1A). We analysed the structural motifs and various domains of MYCT1 using online software SMART, Pfam and CBS Prediction Servers. The predicted domains included two TM domains (amino acid 26–48 and amino acid 68–90), one putative NLS (amino acid 91–114) (Fig. 1B). In addition, we cloned proximal 780 bp (−925 bp/−145 bp) region from HeLa genome as MYCT1 promoter and identified two E‐box (−838 bp/−828 bp and −698 bp/−688 bp) in this region. Overexpression of c‐MYC increased MYCT1 promoter activity to about threefold in HEK293T cells (Fig. 1C). It is consistent with the previous study that c‐MYC binds to the promoter of MYCT1 and regulates its expression 15.

### TM domain is crucial to the cytoplasmic localization of MYCT1

**Figure 2:**
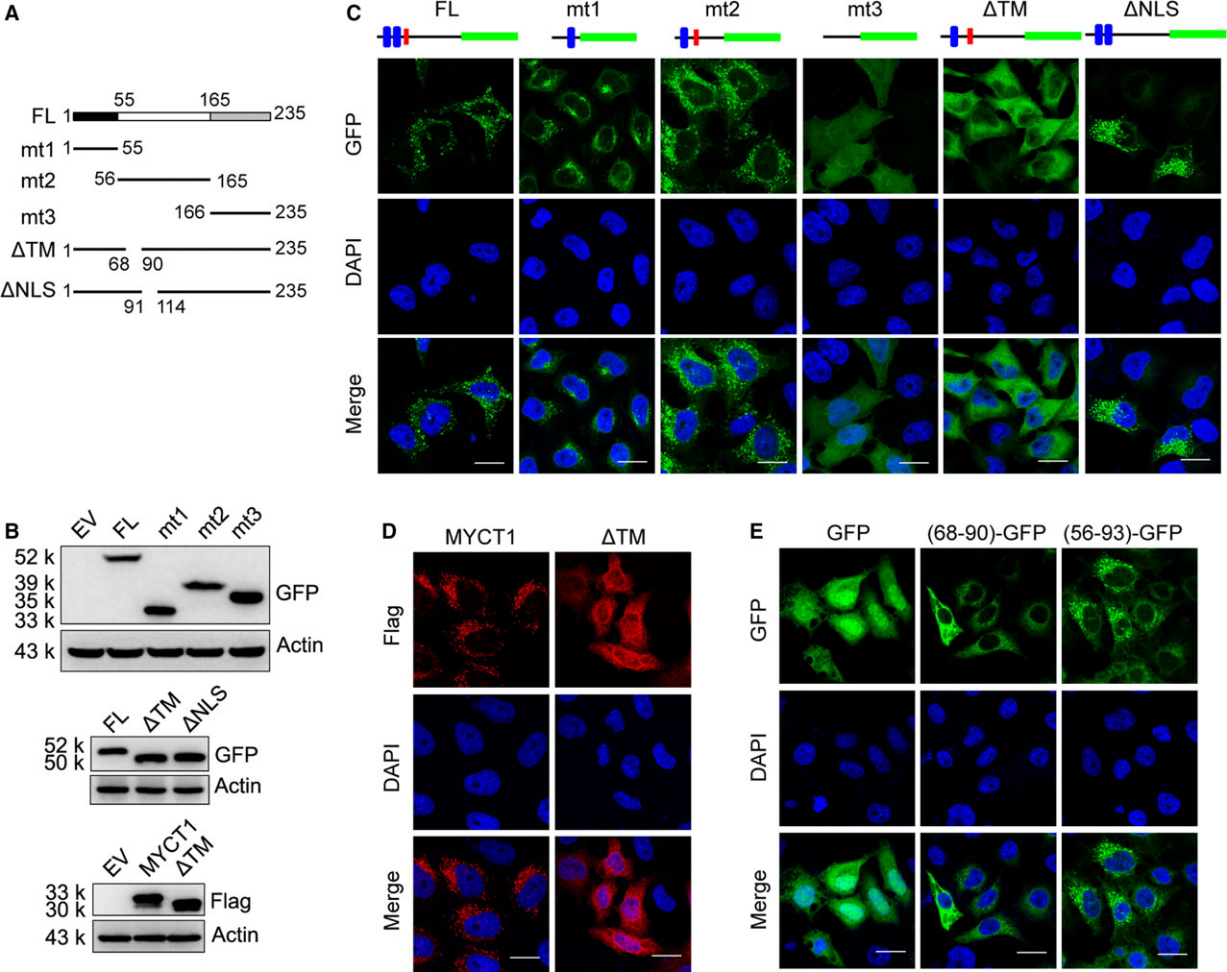
TM domain determines the cytoplasmic localization of MYCT1. (**A**) Schematic diagram of MYCT1 truncation mutants. (**B**) Immunoblot of MYCT1 truncation mutants. (**C** and **D**) Localization of GFP‐fused (**C**) and Flag‐tagged (**D**) MYCT1 wild‐type and truncation mutants in HeLa stable cells as detected by confocal microscopy. (**E**) Localization of GFP‐fused region 68‐90aa or 56‐93aa, respectively, in HeLa stable cells as detected by confocal microscopy; scale bar in C–E, 20 μm.

To observe the localization of MYCT1, we generated a HeLa stable cell line expressing MYCT1‐GFP fusion protein. Confocal microscopy revealed that MYCT1 located granularly in the cytoplasm (Fig. 2C). The localization of MYCT1 in HT‐29 and A549 cells was consistent with that in HeLa cells (Fig. S1A). We next sought to identify which domain of MYCT1 contributed to the granular distribution. Different truncations of MYCT1 fused with GFP were constructed, and stably expressed in HeLa cells (Fig. 2A). All the expression of mutations was detected by immunoblot (Fig. 2B).

Compared with truncation mt1 (containing the first TM domain), mt2 (containing the second TM domain and NLS motif) presented more similar localization as that of full‐length, whereas the mt3 mutant showed completely even distribution (Fig. 2C). Considering the second TM domain is conserved among species, we then focused on it and the NLS motif to identify which one is crucial to the MYCT1 localization. We constructed HeLa stable cell lines expressing GFP‐fused TM deletion (amino acid 68–90) or NLS deletion (amino acid 91–114) mutants respectively. The localization of TM‐deleted mutant was completely changed from punctate distribution to uniform distribution (Fig. 2C, S1B). However, the NLS‐deleted protein did not present an exclusive punctate distribution nor completely diffuse (Fig. 2C). In consideration of the close distance of the TM domain and the NLS domain, we suspected that the NLS deletion might affect TM domain and cause slight localization change. Since GFP is a 26 kD protein, it may affect the localization of fusion protein. We then created stable cell lines expressing Flag epitope‐tagged MYCT1 or the mutant MYCT1(ΔTM) (Fig. 2B), and repeated the confocal microscopy test. The localization of flag epitope‐tagged MYCT1 was consistent with the GFP fusion protein (Fig. 2D). These results demonstrate that TM domain is critical to the localization of MYCT1.

Next, we fused amino acid 68–90 to the N‐terminus of GFP protein as a reporter protein. Majority of (68–90)‐GFP protein localized in cytoplasma in contrast to GFP protein. However, (68–90)‐GFP protein did not present granular distribution like MYCT1‐GFP (Fig. 2E). We speculated that proximal sequences are required to determine the localization of GFP because of spatial structure. So we extended the domain region to 56–93aa, and constructed a (56–93)‐GFP fusion protein. (56–93)‐GFP protein displayed granular distribution (Fig. 2E). These results indicate that the extended TM domain is sufficient to change the localization of GFP reporter from uniform distribution to granular distribution.

### MYCT1 localizes in the cytoplasmic membrane‐associated vesicles

**Figure 3:**
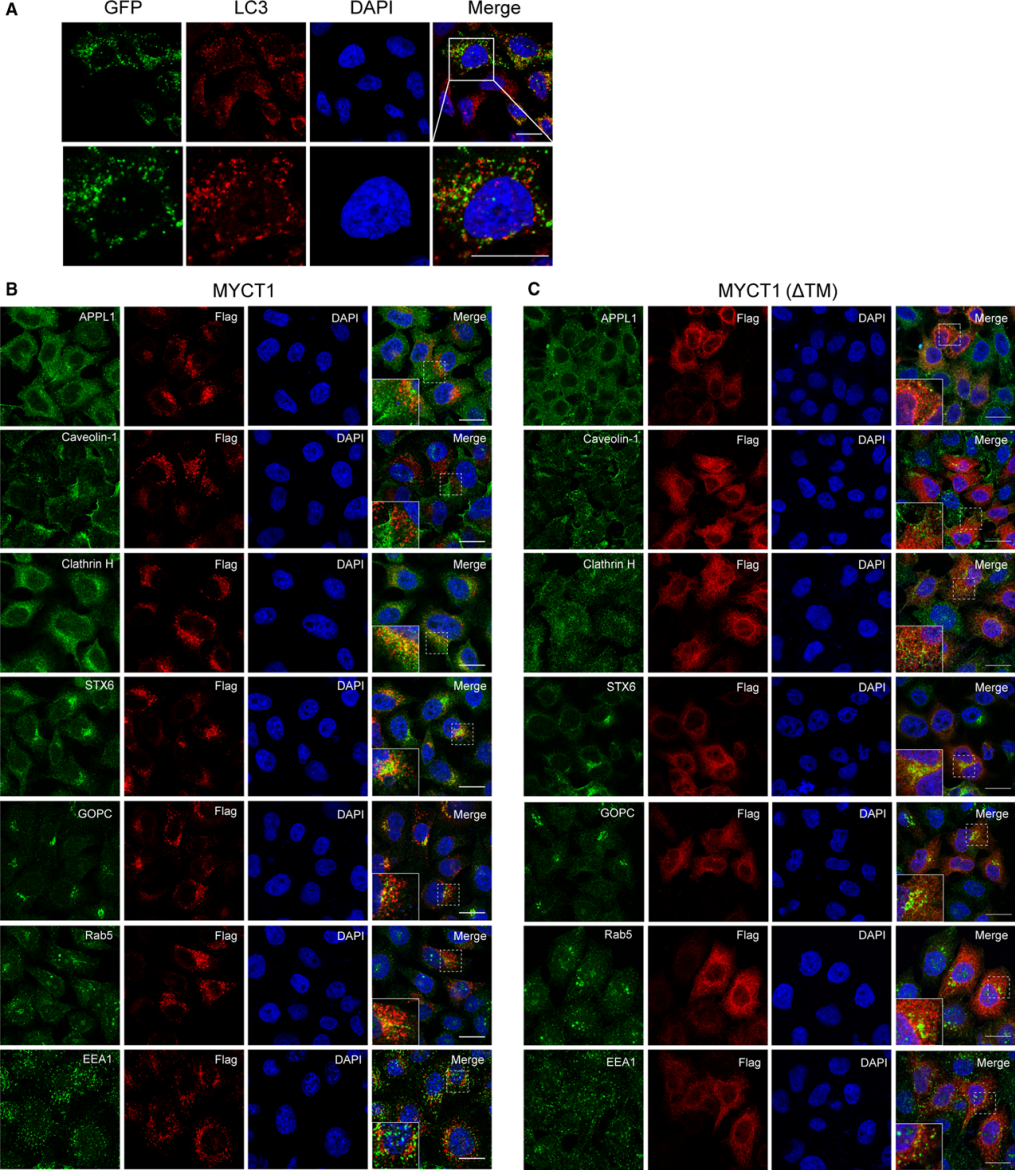
MYCT1 localizes in the cytoplasmic membrane‐associated vesicles and interacts with STX6. (**A**) MYCT1 did not localize to autophagosomal membrane. HeLa cells stably expressing MYCT1‐GFP were starved for 12 hrs, and then immunofluorescence stained with LC3 (red) antibody. The boxed area is shown at a higher magnification (bottom); scale bar, 20 μm. (**B** and **C**) Colocalization of MYCT1 (**B**) or MYCT1(ΔTM) (**C**) and components of vesicle complex in HeLa cells stably expressing Flag‐tagged MYCT1 or Flag‐tagged MYCT1(ΔTM). Antibodies against APPL1, Caveolin‐1, Clathrin H, STX6, GOPC, Rab5 and EEA1 were used; scale bar, 20 μm.

We sought to better detail the subcellular localization of MYCT1. We speculated that MYCT1 distributed in lysosome or membrane vesicle because MYCT1 presented a dot‐like structure. Microtubule‐associated protein 1 light chain 3 alpha (LC3) is involved in the late stage of autophagy. LC3‐I converts to LC3‐II and localizes to autophagosomal membranes when cell is starved. However, we did not observe distinct co‐localization of MYCT1 and LC3‐II in the starved HeLa cells (Fig. 3A), indicating that MYCT1 did not localize to autophagosomal membranes. We next used a series of vesicle trafficking antibodies as markers. Confocal images showed that MYCT1 mainly co‐localized with Clathrin Heavy Chain (Clathrin H) and Syntaxin 6 (STX6) (Fig. 3B), whereas MYCT1(ΔTM) did not colocalize with these markers (Fig. 3C). This indicates that MYCT1 localizes in the cytoplasmic membrane‐associated trafficking vesicles.

### MYCT1 affects tumour cell viability and cell migration

**Figure 4:**
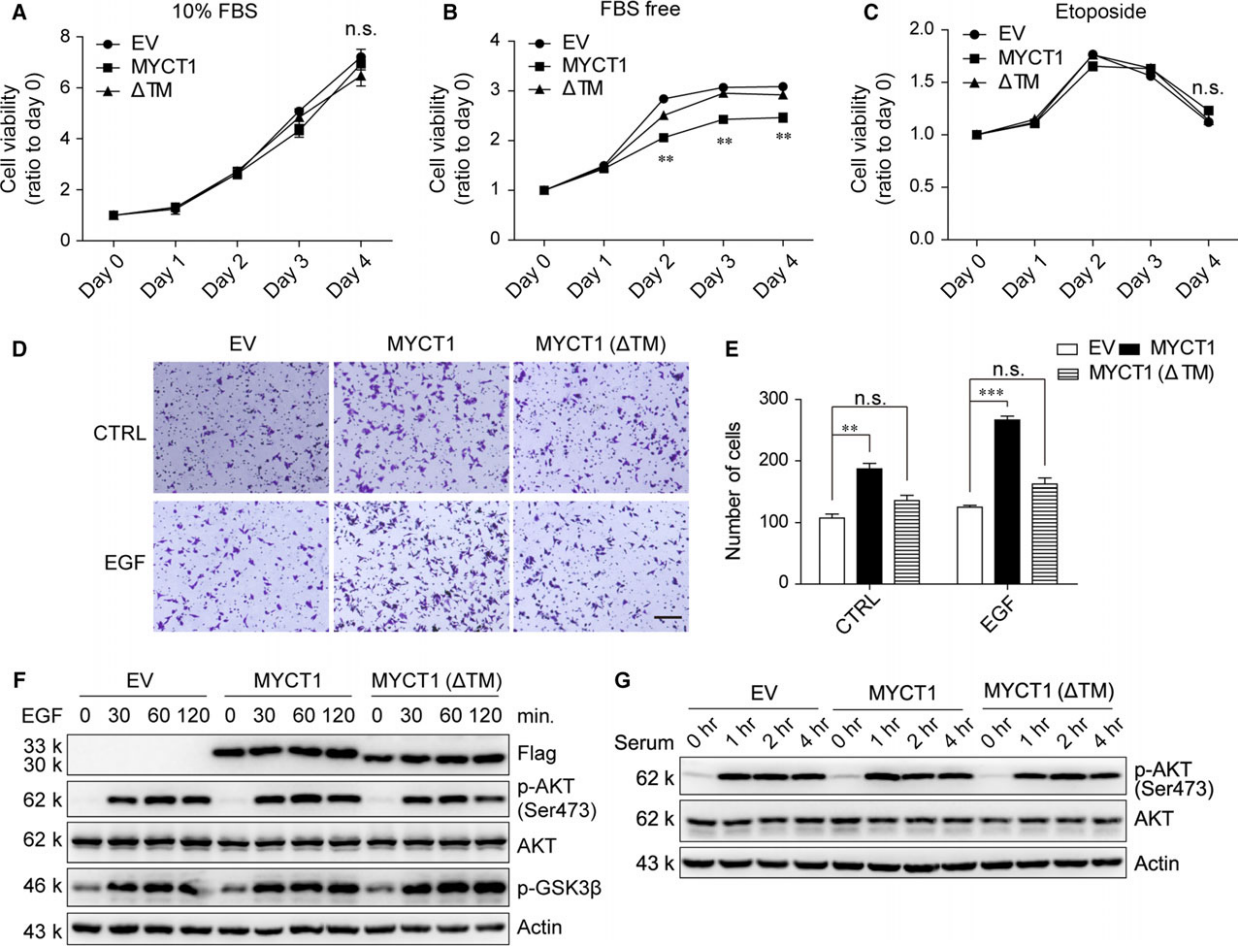
Effects of MYCT1 on tumour cell viability and migration. (**A**–**C**) HeLa cells stably expressing Flag‐tagged MYCT1 and Flag‐tagged MYCT1(ΔTM) were cultured with DMEM containing 10% FBS (**A**), DMEM (**B**), or etoposide (5 μg/ml) in the DMEM containing 10% FBS (**C**) for the indicated time and then analysed by MTT assay. Data are shown as fold change relative to that of day 0. All data shown represent mean ± S.D. (n = 6). (**D**) HeLa cells stably expressing Flag‐tagged MYCT1 or Flag‐tagged MYCT1(ΔTM) were seeded in the migration chamber with 100 ng/ml EGF treatment or not. Cells were stained and counted 24 hrs later; scale bar, 100 μm. (**E**) Quantification of cell migration assay. Four random fields of per treatment were counted. Bar graphs represent mean ± S.D. (n = 4). Experiments were repeated three times. **P < 0.01, ***P < 0.001, n.s., not significant. (**F** and **G**) Cells in (**D**) were pre‐starved for 12 hrs and then treated with 100 ng/ml EGF (**F**) or 10% FBS (**G**) or not for the indicated time. Phosphorylation of AKT was detected by immunoblot.

Published work showed that overexpression of MYCT1 reduced intrinsic viability in the absence of serum and cytokine supplements in monocytes and granulocytes, and enhanced cell survival responses to GM‐CSF 17. We detected the effects of MYCT1 or MYCT1(ΔTM) on cell viability in response to regular medium, serum‐free medium or challenge with chemotherapy drug etoposide. Overexpression of MYCT1 or MYCT1(ΔTM) did not affect HeLa cell viability in regular medium (Fig. 4A) or containing etoposide (Fig. 4C). However, MYCT1 overexpression reduced cell viability responding to serum starvation and MYCT1(ΔTM) did not (Fig. 4B). To examine whether MYCT1 affects tumour cell migration, we performed a transwell migration assay. Overexpression of MYCT1 significantly increased the migration activity about twofold in HeLa stable cells compared with EV‐infected cells. However, overexpression of MYCT1(ΔTM) did not obviously increase the migration activity (Fig. 4D and E). Interestingly, we found EGF accelerated tumour cell migration more greatly in the presence of MYCT1 than EV or MYCT1(ΔTM) (Fig. 4E). As a main indicator of growth factor, we next detected the response of PI3K/AKT signalling pathway. However, the activation of AKT and its target GSK3β induced by EGF or serum was comparable in these stable cells (Fig. 4F and G). So, MYCT1‐mediated migration in response to EGF is independent of PI3K/AKT signalling pathway. We conclude that MYCT1 reduces cell viability in response to growth factor deprivation and promotes cell migration, and TM domain is important to these MYCT1‐mediated functions.

### CKAP4 interacts with MYCT1

**Figure 5:**
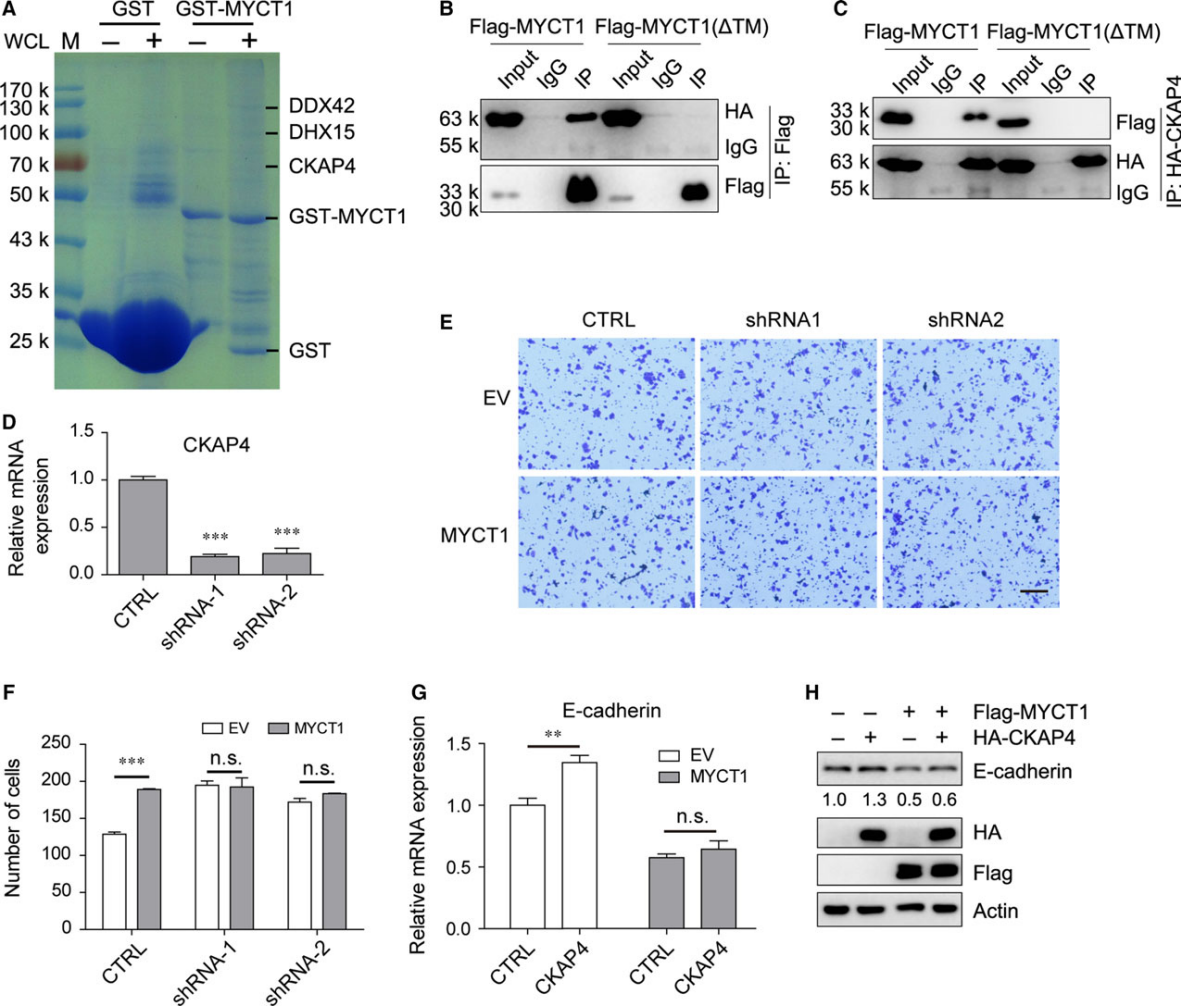
CKAP4 is involved in MYCT1‐mediated tumour migration. (**A**) Recombinant GST‐MYCT1 protein was incubated with HeLa cell lysate. The complex was subjected to SDS‐PAGE and coomassie staining. Specific bands were analysed by MS. (**B** and **C**) HEK293T cells were transfected with Flag‐MYCT1 or Flag‐MYCT1 (ΔTM) expression plasmid along with HA‐CKAP4 expression plasmid. Cell lysates were immunoprecipitated with anti‐HA or anti‐Flag antibodies, respectively, and then immunoblotted with the indicated antibodies. Representative immunoblots in three independent experiments were shown. (**D**) Detection of CKAP4‐shRNA efficiency as measured by quantitative RT‐PCR. (**E**) HeLa stable cells expressing MYCT1 or CKAP4 shRNA were seeded in the migration chamber and cells were stained and counted 24 hrs later. (**F**) Quantitative analysis of the cell migration assay in (**E**). (**G** and **H**) HEK293T cells were transfected with Flag‐MYCT1 expression plasmid or HA‐CKAP4 expression plasmid alone or combined. RNAs and protein were extracted after 36 hrs and E‐cadherin levels were analysed by quantitative RT‐PCR (**G**) and immunoblot (**H**). qRT‐PCR data were normalized to GAPDH gene and are shown as fold change relative to that of control plasmid‐treated cells. Data represent mean ± S.D. (n = 4). Experiments were repeated three times. **P < 0.01, ***P < 0.001, n.s., not significant.

We then performed GST pull‐down assay to look for the proteins that interact with MYCT1. HeLa cell lysate was collected and incubated with purified GST‐tagged MYCT1 protein. The co‐precipitated complex was subjected to SDS‐PAGE and coomassie staining. Three visibly specific bands were pulled down with GST‐MYCT1, and were identified as DDX42, DHX15 and CKAP4, respectively, by MS (Fig. 5A, Table S1). The interaction between MYCT1 and CKAP4 was confirmed by IP analysis in 293T cells transfected with Flag‐MYCT1 and HA‐CKAP4. In addition, MYCT1(ΔTM) did not interact with CKAP4 (Fig. 5B and C). Confocal images showed that MYCT1, but not MYCT1(ΔTM), partly co‐localized with CKAP4 (Fig. S2).

It was reported that CKAP4 plays important roles in several cellular processes, including endoplasmic reticulum anchoring 18 and morphology determination 19, regulation of Dicer function 20, cell proliferation 21 and tumour migration 22, 23. Knockdown of CKAP4 (Fig. 5D) or overexpression of MYCT1 increased HeLa cells migration. However, MYCT1 overexpression did not significantly change CKAP4‐knockdown cells migration (Fig. 5E and F). Overexpression of CKAP4 increased E‐cadherin expression, which is consistent with previous report 22. MYCT1 overexpression abolished the CKAP4‐mediated E‐cadherin up‐regulation (Fig. 5G and H), suggesting that MYCT1 might increase cell migration by inhibiting CKAP4 function.

### A88D mutant changes the localization of MYCT1

**Figure 6:**
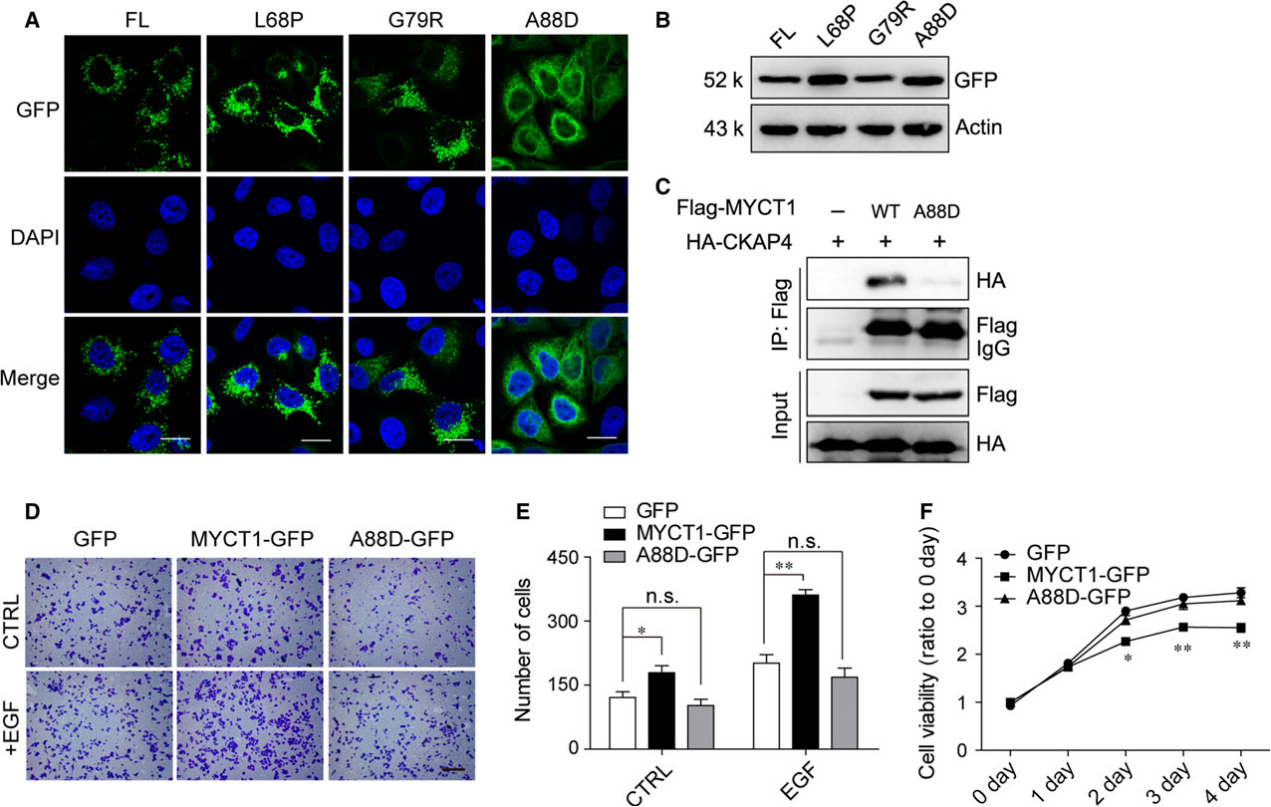
A88D mutant changes the localization and function of MYCT1. (**A**) Localization of GFP‐fused MYCT1 site mutants L68P, G79R and A88D in HeLa stable cells as detected by confocal microscopy; scale bar, 20 μm. (**B**) Detection of different mutants by immunoblot. (**C**) HEK293T cells were transfected with Flag‐MYCT1 or Flag‐MYCT1(A88D) expression plasmid along with HA‐CKAP4 expression plasmid. Cell lysates were immunoprecipitated with anti‐Flag antibody, and then immunoblotted with the indicated antibodies. Representative immunoblots in three independent experiments were shown. (**D**) HeLa cells stably expressing MYCT1‐GFP or A88D‐GFP were seeded in the migration chamber with 100 ng/ml EGF treatment or not. Cells were stained and counted 24 hrs later; scale bar, 100 μm. (**E**) Quantitative analysis of the cell migration assay in (**D**). Four random fields of per treatment were counted. Bar graphs represent mean ± S.D. (n = 4). Experiments were repeated three times. (**F**) Cell viability of stable cells in (**D**) was measured under serum‐deprived conditions by MTT assay. Data are shown as fold change relative to that of day 0. All data shown represent mean ± S.D. (n = 5). *P < 0.05, **P < 0.01. n.s., not significant, *versus* EV group.

To figure out whether MYCT1 protein was mutated in clinical cancer samples, we referred the MYCT1 gene using the Catalogue Of Somatic Mutations In Cancer dataset, and found three point mutations (L68P, G79R and A88D) in the TM region in three clinical cancer patient samples respectively (sample name TCGA‐F4‐6460‐01, TCGA‐G5‐6641‐01, TCGA‐86‐6851‐01). We then examined the localization of these three mutants. Only A88D mutation caused localization change identical to the ΔTM mutant, indicating that Ala88 is indispensable to the localization of MYCT1 (Fig. 6A and B). In addition, the binding between A88D mutant and CKAP4 was very weak (Fig. 6C).

We next evaluated the effect of A88D mutant on tumour cell migration and cell viability. Overexpression of A88D‐GFP mutant did not significantly affect cell migration (Fig. 6D and E), and A88D‐GFP overexpression did not significantly affect cell viability compared with MYCT1‐GFP under serum‐deprived conditions (Fig. 6F). These results indicate that A88D mutant has no effect on cell migration and cell viability, similar to that of MYCT1(ΔTM).

## Discussion

MYCT1 is the target gene of c‐MYC and its murine homologue MT‐MC1 can recapitulate global c‐Myc‐induced phenotypes in animal model 12. It was reported that MT‐MC1 affected cell proliferation, morphology, apoptosis, genomic stability and differentiation 13. Liddiard *et al*. found that overexpression of MYCT1 reduced acute myeloid leukaemia cell viability in the culture medium without serum, but did not change cell differentiation. All these studies indicate that MYCT1 may possess multiple functions. However, basic knowledge of MYCT1, such as the localization, is still needed to be elucidated.

Previous study showed that the murine homologue of MT‐MC1 localized in the nucleus 13. In this study, we identified that human MYCT1 localized predominantly to the cytoplasmic vesicle complex although MYCT1 protein contains a putative NLS motif, which usually is a nuclear import signal. Our work proved that the putative NLS motif is not required for the localization of MYCT1. We were not able to confirm the subcellular localization of endogenous MYCT1 in several tumour cell lines, including HeLa, 293T, SW480, HCT116 and A549 cells, using commercial or homemade MYCT1 antibody (data not shown). We assume that the MYCT1 protein level is too low to be detected in our system. The MYCT1 mRNA level detected by quantitative RT‐PCR indicated its low expression (data not shown). The localization of exogenous MYCT1 in HeLa, HT‐29 and A549 cells was consistent (Fig. 2C and Fig. S1).

We found that the TM domain (68–90aa) is crucial to the localization of MYCT1 (Fig. 2). Fu *et al*. reported that there is another MYCT1 transcript variant (MYCT1‐TV), which encodes a protein beginning from N‐terminal Met49 of MYCT1 protein 15. However, no work confirmed the MYCT1 or MYCT1‐TV at the protein levels so far. The first TM domain (26–48aa) does not exist in MYCT1‐TV or other species. This is another reason that we thought the second TM domain (68–90aa) is more important than the first one to the function of MYCT1. In addition, our work first identified Ala88, which mutates into aspartic acid (Asp) in a clinical lung cancer patient sample, contributed to the subcellular localization of MYCT1 (Fig. 6A). This suggested that localization change in MYCT1 might participate in tumorigenesis.

Overexpression of MYCT1 did not change the expression and the cellular localization of β‐catenin as well as the expression of classic Wnt target genes, such as AXIN2, c‐MYC, CCND1, DKK‐1 and SFRP1 (data not shown). We proposed that MYCT1 was not able to mimic the function of c‐MYC in activation of Wnt signalling pathway. We also found that the MYCT1 promoter activity is not regulated by activation of Wnt signalling, although c‐MYC is a target of Wnt signalling. Liddiard *et al*. recently reported that MYCT1 is a target gene of RUNX1‐ETO, prompting that MYCT1 might be regulated by multifactors in addition to c‐MYC. Additional studies are required to explore the molecular mechanism of MYCT1 in Wnt signalling pathway.

Our work first identified three MYCT1 interacting proteins. All of them are related to RNA processing 20, 24, 25. This might be one of the reasons that MYCT1 is able to recapitulate multiple c‐MYC‐related phenotypes. Previous studies showed that EMT and EGFR signalling were inhibited by CKAP4. We found that overexpression of MYCT1 abolished CKAP4‐mediated up‐regulation of E‐cadherin (Fig. 5G and H) and CKAP4 interference‐induced tumour cell migration (Fig. 5E and F). We suppose that MYCT1 might enhance the tumour cell migration through inhibiting CKAP4 function. In addition, change in E‐cadherin level is probable not the main reason of CKAP4 interference‐induced cell migration, because CKAP4 depletion increases migration of HeLa cells, which is an E‐cadherin negative cell line 26.

Recently, Masiero *et al*. reported that the G‐protein‐coupled receptor ELTD1 and MYCT1 are highly expressed in multiple types of tumour‐associated endothelial cells 27. Using the online database (Search Tool for the Retrieval of Interacting Genes), we found that MYCT1 is predicted to functionally interact with ELTD1 and EMCN (endomucin). ELTD1 and EMCN are TM proteins located on cytoplasmic membrane and function in tumour migration and angiogenesis. Our work showed that MYCT1 localizes in the cytoplasmic membrane‐vesicle complex. The potential correlation between MYCT1 and ELTD1 or EMCN implied that MYCT1 might function through affecting the trafficking of cytoplasmic vesicles. Future studies of MYCT1 will explore the protein interaction between MYCT1 and the angiogenesis‐associated proteins.

## Conflicts of interest

The authors declare no competing interests.

## Supporting information


**Figure S1** HT‐29 and A549 cells were infected with lentivirus expressing MYCT1‐GFP (A) or MYCT1(ΔTM)‐GFP (B), and detected by confocal microscopy (green). Scale bar, 20 μm.

**Figure S2** Colocalization of WT‐MYCT1 or MYCT1(ΔTM) and CKAP4 was detected in HeLa cells by immunofluorescence. Scale bar, 20 μm. Right, quantification of colocalization of WT‐MYCT1 or MYCT1(ΔTM) and CKAP4.

**Table S1** MS data of MYCT1 interacting proteins.
