# Supplementary figures and images for: Transmembrane domain is crucial to the subcellular localization and function of Myc target 1
Source: J Cell Mol Med. 2015 Dec 29;20(3):471–81. doi: 10.1111/jcmm.12747 (PMC4759468; doi:10.1111/jcmm.12747)

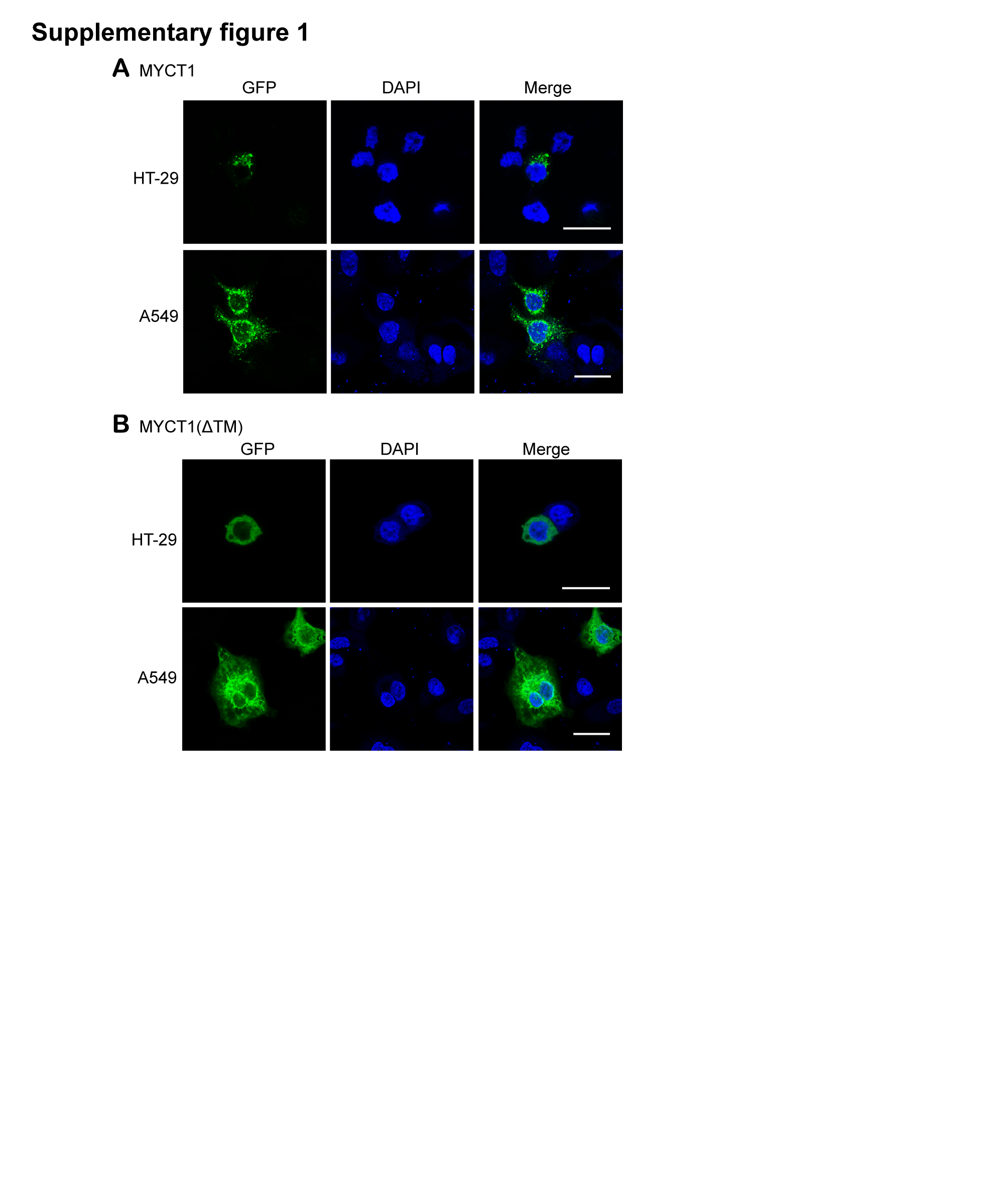

Supplement: Supplementary file 1 — Figure S1 HT‐29 and A549 cells were infected with lentivirus expressing MYCT1‐GFP (A) or MYCT1(ΔTM)‐GFP (B), and detected by confocal microscopy (green). Scale bar, 20 μm. [file JCMM-20-471-s001.tif]

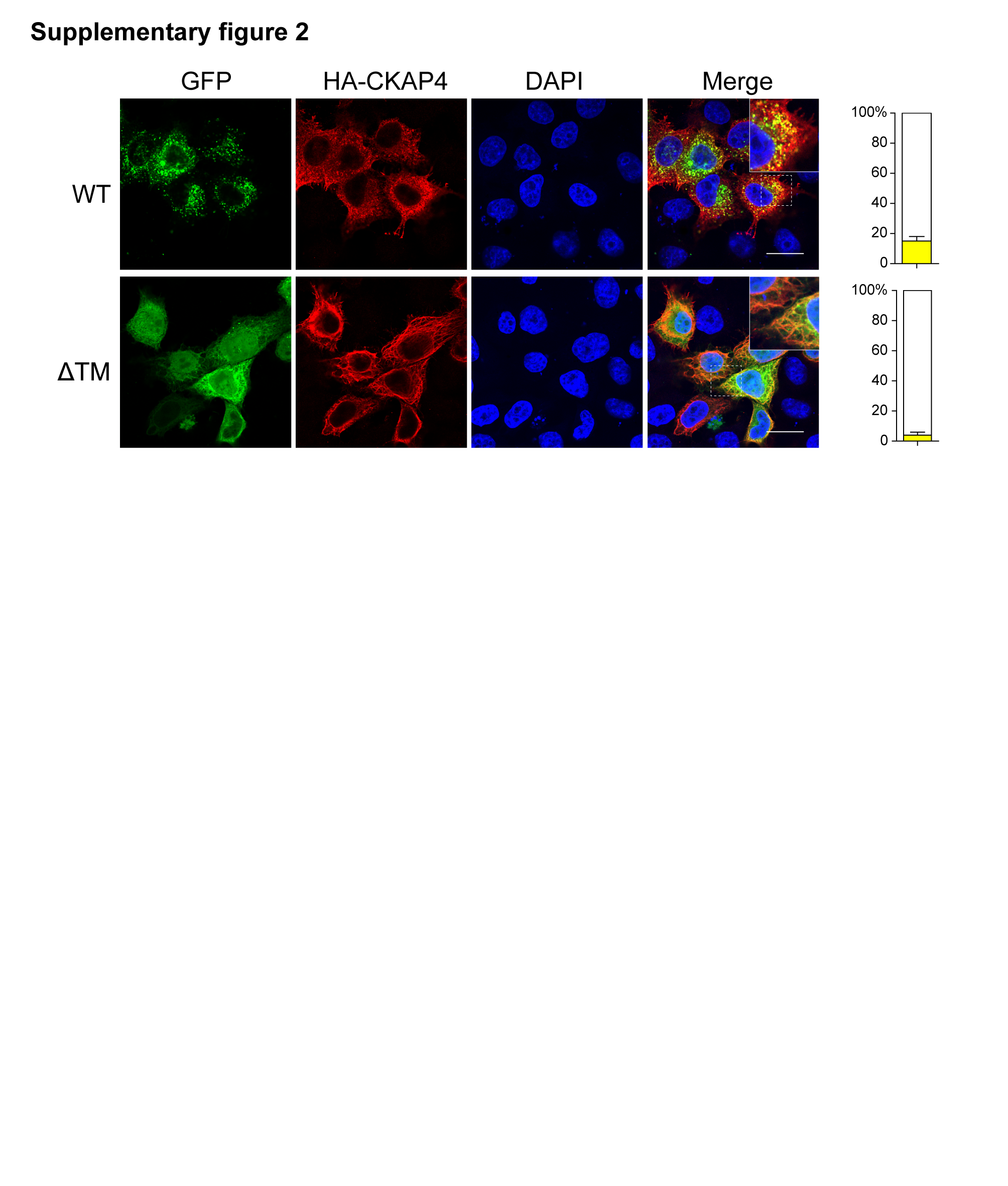

Supplement: Supplementary file 2 — Figure S2 Colocalization of WT‐MYCT1 or MYCT1(ΔTM) and CKAP4 was detected in HeLa cells by immunofluorescence. Scale bar, 20 μm. Right, quantification of colocalization of WT‐MYCT1 or MYCT1(ΔTM) and CKAP4. [file JCMM-20-471-s002.tif]
